# Clinical benefits of PD-1 inhibitors in specific subgroups of patients with advanced esophageal squamous cell carcinoma: a systematic review and meta-analysis of phase 3 randomized clinical trials

**DOI:** 10.3389/fimmu.2023.1171671

**Published:** 2023-05-02

**Authors:** Yao Lu, Wenkang Wang, Feng Wang

**Affiliations:** ^1^ Department of Oncology, The First Affiliated Hospital of Zhengzhou University, Zhengzhou, China; ^2^ Department of Breast Surgery, The First Affiliated Hospital of Zhengzhou University, Zhengzhou, China

**Keywords:** PD-1 inhibitor, esophageal squamous cell carcinoma, subgroup analyses, immunotherapy, survival benefit

## Abstract

**Purpose:**

In recent years, a number of clinical trials have shown that programmed death 1 (PD-1) inhibitors offer significant survival benefits in patients with esophageal squamous cell carcinoma (ESCC). We conducted a meta-analysis to explore the antitumour efficacy of PD-1 inhibitor-based therapy in specific subgroups of patient with advanced ESCC.

**Methods:**

We searched for eligible studies from the PubMed, Embase, Web of Science, Cochrane Library databases and conference abstracts. The indicators related to survival outcomes were extracted. The pooled hazard ratios (HRs) for overall survival (OS), progression-free survival (PFS) and duration of response (DOR) and the pooled odds ratio (OR) for objective response rate (ORR) were calculated to evaluate the efficacy of PD-1 inhibitor-based therapy in ESCC. Data regarding treatment lines, treatment regimens, programmed death ligand 1 (PD-L1) status, baseline demographic and disease characteristics were extracted. Subgroup analyses were conducted in specific populations of ESCC patients. The Cochrane risk of bias tool and sensitivity analysis were used to assess the quality of the meta-analysis.

**Results:**

Eleven phase 3 randomized controlled trials (RCTs) involving 6267 patients with ESCC were included in this meta-analysis. Compared with standard chemotherapy, PD-1 inhibitor-based therapy provided benefits in terms of OS, PFS, ORR, and DOR in all populations, the first-line treatment group, the second-line treatment group, the immunotherapy group, and the immunochemotherapy group. Although a limited PFS benefit was observed in second-line treatments and immunotherapy alone, PD-1 inhibitor-based therapy still reduced the risk of disease progression or death. Patients with high PD-L1 expression had a better OS benefit than those with low PD-L1 expression. The HR for OS favoured PD-1 inhibitor-based therapy over standard chemotherapy for all prespecified clinical subgroups.

**Conclusions:**

Compared with standard chemotherapy, PD-1 inhibitor-based therapy exhibited clinically meaningful benefits in patients with ESCC. Survival benefits were better in patients with high PD-L1 expression than in those with low PD-L1 expression, suggesting that the PD-L1 expression level can be used as a predictor of survival benefit from PD-1 inhibitor therapy. PD-1 inhibitor-based therapy provided a consistent benefit in reducing the risk of death according to prespecified subgroup analyses of clinical characteristics.

## 1 Introduction

Esophageal cancer (EC) is one of the most common malignant tumours in the world. The incidence and mortality of EC rank seventh (604,100 new cases) and sixth (544,076 deaths), respectively (1). Esophageal squamous cell carcinoma (ESCC) is more common in eastern Asia, eastern and southern Africa, and southern Europe, whereas esophageal adenocarcinoma (EAC) is more common in North America and other parts of Europe (2, 3). Patients with ESCC usually present with advanced esophageal obstruction symptoms at diagnosis (4, 5). Based on statistical data, the prognosis of advanced ESCC is always poor, with a 5-year survival rate of approximately 15–25% (6). Standard chemotherapy regimens for advanced ESCC have been explored for decades. Combined chemotherapy regimens, such as paclitaxel plus cisplatin (TP) or cisplatin plus 5-fluorouracil (CF), are standard first-line treatments for ESCC (7, 8). Taxane or irinotecan monotherapy is often used as second-line treatments for advanced ESCC (9–11). However, the overall survival (OS) associated with first-line treatments is less than one year, and that associated with second-line treatments is shorter at only 6-8 months, suggesting that the chemotherapy regimens for advanced ESCC may have reached a bottleneck (12). In recent years, new treatment strategies for advanced ESCC have been explored. Overall, the efficacy of targeted therapy in advanced ESCC remains unclear and needs to be further explored (13).

Immunotherapy has been recognized as a new and effective therapeutic strategy for various types of cancers (14). Programmed cell death protein 1 (PD-1) immune checkpoint inhibitors can specifically bind to PD-1 and block the PD-1/programmed death ligand-1 (PD-L1) signalling pathway to restore the killing function of efficacious immune cells and inhibit tumour growth (15, 16). PD-1 inhibitor-based therapy has led to remarkable responses and clinical benefits in a variety of malignant tumours, including advanced ESCC (17–19). Some studies have shown that PD-L1 is abundantly expressed in ESCC, which suggests that patients with ESCC may benefit from PD-1 inhibitor-based therapy (20). KEYNOTE-181 and ATTRACTION-3 established PD-1 inhibitors as the standard second-line treatment for advanced ESCC (21, 22). KEYNOTE-590 and CheckMate 648 also confirmed that the combination of PD-1 inhibitors and chemotherapy was superior to chemotherapy alone in the first-line treatment of advanced ESCC (23, 24). PD-1 inhibitor monotherapy as a second-line or subsequent treatment and PD-1 inhibitor therapy plus chemotherapy as a first-line treatment are recommended by the National Comprehensive Cancer Network (NCCN). PD-1 inhibitors, including pembrolizumab, nivolumab, camrelizumab, sintilimab and so on, are commonly used in the treatment of advanced ESCC, and clinical trials of other PD-1 inhibitors are also underway.

However, some patients have primary resistance to PD-1 inhibitors and even develop hyperprogressive disease (HPD). The clinical diagnostic criteria for HPD vary between studies, and current criteria for HPD include treatment failure within less than 2 months, an increase in tumour burden of more than 50%, an acceleration in tumour growth kinetics, and a more than threefold increase in tumour progression within 2 months of immunotherapy initiation compared with that on pretreatment imaging (25, 26). Therefore, it is important to identify patients with ESCC who can benefit from immunotherapy.

In some clinical trials, greater clinical benefits in patients with high PD-L1 expression versus those with lower PD-L1 expression have been reported, reinforcing the use of PD-L1 expression to screen patients (27, 28). A study showed that the treatment response to nivolumab was correlated with the expression level of PD-L1 in tumour tissue, 36% of PD-L1 positive patients responded to the treatment, while none had an objective response in PD-L1 negative solid tumours (29). In advanced ESCC, CheckMate 648 found that no obvious survival benefit was observed in the combined immunotherapy group with a PD-L1 tumour proportion score (TPS) <1% (23). KEYNOTE-181 showed that pembrolizumab monotherapy provided a clinically meaningful survival benefit as second-line therapy for advanced ESCC patients with a PD-L1 combined positive score (CPS) ≥10, but the benefit was not observed in CPS <10 (22). The application of PD-1 inhibitors in advanced ESCC also varies from country to country. In terms of first-line treatment, the European Medicines Agency (EMA) approved pembrolizumab for the first-line treatment of ESCC with PD-L1 CPS ≥10, and the Food and Drug Administration (FDA) and National Medical Products Administration (NMPA) approved pembrolizumab as the first-line treatment for ESCC regardless of the PD-L1 expression level. In terms of second-line treatments, pembrolizumab has been approved by the FDA and NMPA for advanced ESCC patients with PD-L1 CPS ≥10. Nivolumab has been approved by the FDA, EMA, and NMPA for the second-line treatment of advanced ESCC regardless of PD-L1 expression status. Therefore, whether the expression level of PD-L1 in ESCC can affect the efficacy of immunotherapy and whether PD-L1 negative ESCC population can benefit from PD-1 inhibitor therapy remain to be explored. The results of some studies have shown that the efficacy of PD-1 inhibitors in earlier treatment lines was better than that in later lines, suggesting that the timing of treatment may be related to the efficacy of PD-1 inhibitors (30–32). An impact of population differences on the efficacy of PD-1 inhibitors has also been reported (33–35). Therefore, based on the current results and subgroup analysis data, we conducted a meta-analysis to observe the benefit of immunotherapy in different populations with ESCC. Importantly, we exclusively focused on phase 3 randomized clinical trials (RCTs) of PD-1 inhibitors in patients with advanced ESCC.

## 2 Materials and methods

### 2.1 Search strategy

We searched for articles from PubMed, Embase, Web of Science, and Cochrane Library. Conference abstracts from the European Society of Medical Oncology (ESMO) and American Society of Clinical Oncology (ASCO) were also searched. The latest search was Dec 1, 2022. The keywords for the search were as follows: (esophageal OR esophagus OR oesophagus OR oesophageal) AND (squamous cell carcinoma OR cancer OR carcinoma OR neoplasm OR neoplasms) AND (PD-1 OR PD-1 inhibitor OR Immune checkpoint inhibitor OR immunotherapy OR Nivolumab OR Opdivo OR Pembrolizumab OR Lambrolizumab OR Atezolizumab OR Camrelizumab OR SHR-1210 OR Tislelizumab OR Toripalimab OR JS001 OR Sintilimab OR Serplulimab) AND (clinical trial OR clinical study). The search strategy was evaluated independently by two authors according to the Preferred Reporting Items for Systematic review and Meta-Analysis (PRISMA).

### 2.2 Study selection

The search results were first evaluated by two independent authors to identify potentially relevant studies. Qualified articles were selected according to the following inclusion criteria: (1) phase 3 RCTs in advanced (recurrent or metastatic) ESCC; (2) random assignment of PD-1 inhibitor-based therapy or chemotherapy; and (3) available details of survival outcomes, such as OS, progression-free survival (PFS), objective response rate (ORR) and duration of response (DOR). Phase 2 or phase 1 trials were excluded. Reviews, cases, or retrospective studies were also excluded. Literature screening was performed independently by two authors according to the criteria, and disagreements were resolved by consultation with all authors.

### 2.3 Data extraction and quality assessment

First, the following information about study characteristics was extracted: the clinical trial name, year of publication, first author’s name and number of patients. Second, the indicators related to survival outcomes were extracted: OS, PFS, ORR, and DOR. Finally, subgroup-related information was extracted: treatment line, treatment regimen, PD-L1 status, age, sex, Eastern Cooperative Oncology Group Performance Status (ECOG PS), region, liver metastasis, previous radiotherapy, and number of organs with metastases. The risk assessment was conducted according to the Cochrane risk of bias tool. The robustness and reliability of the combined outcomes were assessed by sensitivity analysis. Data extraction and quality assessment were performed independently by two authors.

### 2.4 Statistical analysis

To evaluate the efficacy of PD-1 inhibitors in ESCC, the pooled hazard ratios (HRs) and 95% confidence intervals (CIs) for OS, PFS and DOR and the pooled odds ratio (OR) and 95% CI for ORR were calculated. The primary objective of the meta-analysis was to assess the differential effect of PD-1 inhibitor-based therapy on survival benefits across distinct subgroups. Fixed-effects (I^2^ ≤ 50%) or random-effects (I^2^ > 50%) models were applied according to differences in heterogeneity between trials. Review Manager 5.3 (Cochrane Collaboration, Oxford, UK) and Stata 12.0 (Stata Corporation) were used for the meta-analysis. P < 0.05 was considered statistically significant. The statistical analyses were conducted independently by two authors. All discrepancies were resolved by discussion and agreement among the authors.

## 3 Results

### 3.1 Literature selection process and search results

**Figure 1:**
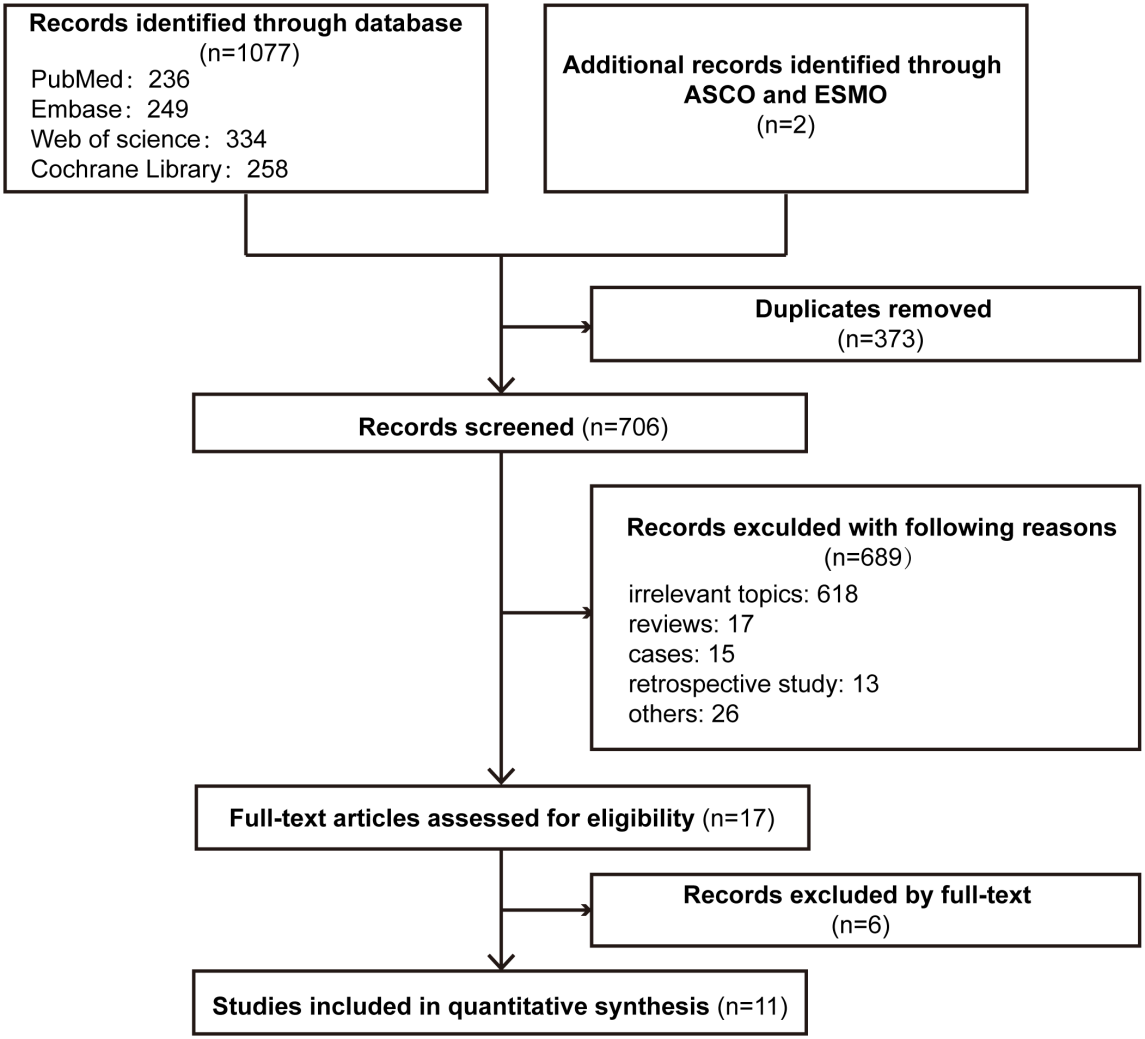
Flowchart of the study selection process for the meta-analysis.

The PRISMA flowchart of the literature selection process for this meta-analysis is shown in **Figure 1**. In total, our search revealed 1079 relevant records, including conference abstracts. A total of 373 records were excluded because of duplication, and 689 records were excluded for other reasons. Seventeen studies were assessed for eligibility, and 11 studies were included in the final analysis (21–24, 36–42).

The characteristics of the included clinical trials are shown in **Table 1**. Eleven studies were phase 3 RCTs, and a total of 6267 patients with ESCC were enrolled. Nine studies included patients with ESCC (21, 23, 36–42), and KEYNOTE-180 and KEYNOTE-590 included patients with ESCC and EAC (22, 24). Data related to ESCC were extracted. Four studies involved second-line treatments (21, 22, 36, 37), and 7 studies involved first-line treatments (23, 24, 38–42). In four studies, the antitumour activity of immunotherapy alone versus chemotherapy (21, 22, 36, 37) was evaluated, and in 6 studies, efficacy was compared between immunotherapy plus chemotherapy (immunochemotherapy) and chemotherapy (24, 38–42). In the CheckMate 648 trial, patients were randomly assigned to receive immunochemotherapy, immunotherapy alone, or chemotherapy alone (23). According to information in CheckMate 648 trial, CheckMate 648-1 was the comparison of nivolumab plus chemotherapy with chemotherapy, and CheckMate 648-2 was the comparison of nivolumab plus ipilimumab with chemotherapy.

**Table 1:** Characteristics of the studies included in the meta-analysis.

Clinal Trials	Line	Treatment Regimen	Patient Number	mOS (month)	HR for OS (95%CI)	mPFS (month)	HR for PFS (95%CI)	ORR (%)
ATTRACTION-3	Second	nivolumab	210	10.9	0.77 (0.62-0.96)	1.7	1.08 (0.87-1.34)	19
		PTX or DTX	209	8.4		3.4		22
KEYNOTE-181	Second	pembrolizumab	198	8.2	0.78 (0.63-0.96)	2.2	0.92 (0.75-1.13)	16.7
		PTX or DTX or CPT-11	203	7.1		3.1		7.4
ESCORT	Second	camrelizumab	228	8.3	0.71 (0.57-0.87)	1.9	0.69 (0.56-0.86)	20.2
		DTX or CPT-11	220	6.2		1.9		6.4
RATIONALE 302	Second	tislelizumab	256	8.6	0.70 (0.57-0.85)	1.6	0.83 (0.67-1.01)	20.3
		PTX or DTX or CPT-11	256	6.3		2.1		9.8
CheckMate 648	First	nivolumab+CF	321	13.2	0.74 (0.58-0.96)	5.8	0.81 (0.64-1.04)	47
		nivolumab +ipilimumab	325	12.7	0.78 (0.62-0.98)	2.9	1.26 (1.04-1.52)	28
		CF	324	10.7		5.6		27
KEYNOTE 590	First	pembrolizuma+CF	274	12.6	0.72 (0.6-0.88)	6.3	0.65 (0.54-0.78)	43.8
		placebo+CF	274	9.8		5.8		31
ESCORT-1st	First	camrelizumab+TP	298	15.3	0.7 (0.56-0.88)	6.9	0.56 (0.46-0.68)	72.1
		placebo+TP	298	12		5.6		62.1
ORIENT-15	First	sintilimab+TP/CF	327	16.7	0.63 (0.51-0.78)	7.2	0.56 (0.46-0.68)	66
		placebo+TP/CF	332	12.5		5.7		45
JUPITER-06	First	toripalimab+TP	257	17	0.58 (0.43-0.78)	5.7	0.58 (0.46-0.74)	69.3
		placebo+TP	257	11		5.5		52.1
RATIONALE 306	First	tislelizumab+TP/CF	326	17.2	0.66 (0.54-0.80)	7.3	0.62 (0.52- 0.75)	63.5
		placebo+TP/CF	323	10.6		5.6		42.4
ASTRUM-007	First	serplulimab+CF	368	15.3	0.68 (0.53-0.87)	5.8	0.60 (0.48-0.75)	57.6
		placebo+CF	183	11.8		5.3		42.1

OS, overall survival; PFS, progression-free survival; HR, hazard ratio; PTX, paclitaxel; DTX, docetaxel; CPT-11, irinotecan; CF, cisplatin plus 5-fluorouracil; TP, paclitaxel plus cisplatin.

### 3.2 Treatment lines

Overall, compared with standard chemotherapy, PD-1 inhibitor-based therapy provided significant benefits in terms of OS (HR: 0.71; 95% CI: 0.66-0.75), PFS (HR: 0.74; 95% CI: 0.63-0.86), ORR (OR: 1.91; 95% CI: 1.57-2.33), and DOR (HR: 0.56; 95% CI: 0.47-0.66) (**Figure S1**).

**Figure 2:**
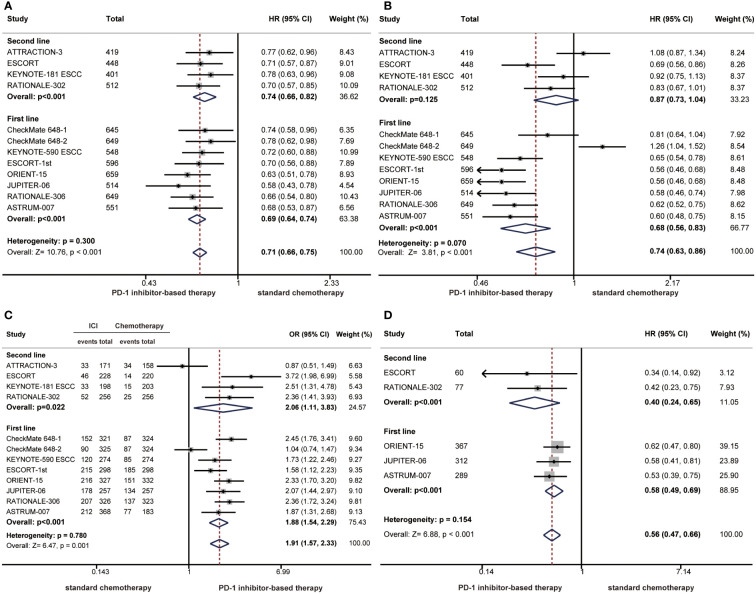
Forest plots of subgroup analysis by treatment lines comparing overall survival **(A)**, progression-free survival **(B)**, objective response rate **(C)**, and duration of response **(D)** in patients who received PD-1 inhibitor-based therapy versus chemotherapy. (HR, hazard ratio; OR, odds ratio; CI, confidence interval; PD-1, programmed cell death 1).

We also performed subgroup analyses according to treatment lines. Compared with chemotherapy, PD-1 inhibitor-based therapy significantly prolonged OS as a first-line treatment (HR: 0.69; 95% CI: 0.64-0.74) or second-line treatment (HR: 0.74; 95% CI: 0.66-0.82) in patients with ESCC (**Figure 2A**). The pooled HR of PFS showed that PD-1 inhibitor-based therapy reduced the risk of disease progression in the first-line treatment (HR: 0.68; 95% CI: 0.56-0.83), but the PFS benefit was limited in the second-line treatment (HR: 0.87; 95% CI: 0.73-1.04) (**Figure 2B**). The ORR was significantly higher in the PD-1 inhibitor group than in the chemotherapy group in first-line (OR: 1.88; 95% CI: 1.54-2.29) and second-line treatments (OR: 2.06; 95% CI: 1.11-3.83) (**Figure 2C**). PD-1 inhibitor-based therapy offered a favourable DOR benefit in first-line (HR: 0.58; 95% CI: 0.49-0.69) and second-line treatments (HR: 0.40; 95% CI: 0.24-0.65) (**Figure 2D**).

The survival benefit was similar in first-line treatments and second-line treatments as the difference did not reach statistical significance (OS: P = 0.300; PFS: P = 0.070; ORR: P=0.780; DOR: P=0.154) (**Figure 2**). Notably, there were limited benefits of PFS in second-line treatments, but better PFS benefits were observed with first-line treatments. The difference in PFS benefits across treatment line subgroups was observed as a near-significant trend (P = 0.070) (**Figure 2B**).

### 3.3 Treatment regimens

**Figure 3:**
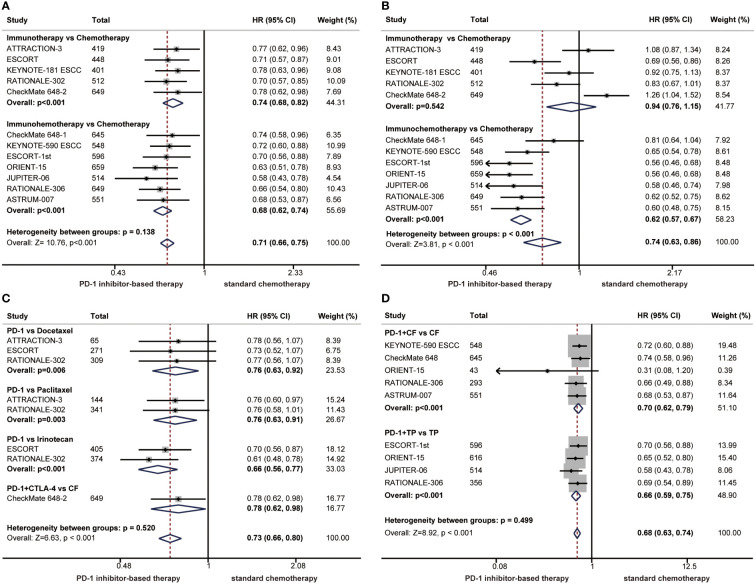
Forest plots of subgroup analysis by treatment regimens comparing benefits of overall survival **(A)** and progression-free survival **(B)**. Subgroup analyses according to chemotherapy regimens comparing overall survival in patients who received immunotherapy alone versus chemotherapy **(C)** or in patients who received immunochemotherapy versus chemotherapy **(D)**. (HR, hazard ratio; CI, confidence interval; PD-1. programmed cell death 1; CTLA-4, cytotoxic T lymphocyte-associated antigen-4).

Currently, a variety of immunotherapy regimens have been explored in phase 3 clinical trials of ESCC, such as immunotherapy alone or immunochemotherapy. Therefore, we performed subgroup analyses according to treatment regimens. The HR of the OS benefit was 0.74 (95% CI: 0.68-0.82) in patients receiving immunotherapy compared with 0.68 (95% CI: 0.62-0.74) in patients receiving immunochemotherapy, and there was no significant difference between the two groups (P = 0.138) (**Figure 3A**). In terms of PFS, the risk of disease progression was reduced by 38% in the immunochemotherapy group compared with 6% in the immunotherapy alone group. The PFS benefit was limited in the immunotherapy alone group, and differences between immunotherapy and immunochemotherapy were significant, demonstrating that immunochemotherapy provided a higher PFS benefit (P < 0.001) (**Figure 3B**).

In the clinical trials of immunotherapy alone versus chemotherapy, chemotherapy regimens have varied, so we performed subgroup analyses according to chemotherapy regimens in patients with ESCC. A significant improvement in OS was found among patients treated with immunotherapy alone compared with those treated with chemotherapy, regardless of chemotherapy regimens. No significant differences were observed in the subgroup of chemotherapy regimens (P = 0.520) (**Figure 3C**). The chemotherapy regimens combined with immunotherapy are usually TP or CF. The combination of PD-1 inhibitors with TP or CF significantly improved OS compared with chemotherapy alone, and there were no significant differences between the CF group and TP group (P = 0.499), demonstrating that both chemotherapy regimens can be combined with immunotherapy for patients with ESCC (**Figure 3D**).

### 3.4 PD-L1 status

**Figure 4:**
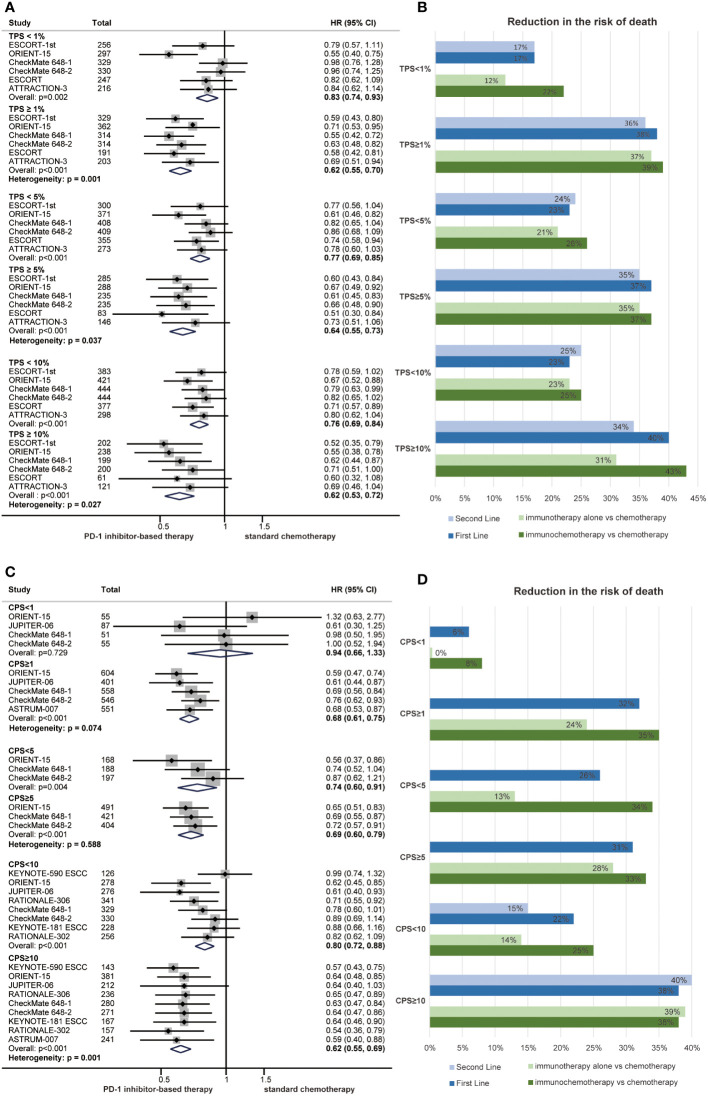
Forest plots of overall survival by subgroup analyses of PD-L1 TPS **(A)** and PD-L1 CPS **(C)**. The reduced risk of death from PD-1 inhibitor-based therapy by subgroup analyses of PD-L1 TPS **(B)** and PD-L1 CPS **(D)** in first-line treatments, second-line treatments, immunotherapy and immunochemotherapy. (HR, hazard ratio; CI, confidence interval; PD-1, programmed cell death 1; TPS, tumour proportion score; CPS, combined positive score).

We conducted a summary analysis of the PD-L1 immunohistochemistry kits and antibodies used in the RCTs included in the meta-analysis, and the proportion of patients with positive PD-L1 expression was similar among groups (**Supplementary Table 1**). Subgroup analyses were performed according to PD-L1 status to observe the relationship between PD-L1 expression level and the efficacy of PD-1 inhibitors in ESCC. TPS and CPS are the most commonly used PD-L1 scoring methods. The HR for OS favoured PD-1 inhibitor-based therapy over chemotherapy in all prespecified subgroups, but the OS benefit was limited in the PD-L1 CPS<1 subgroup (**Figures 4A, C**). Notably, we demonstrated that PD-L1 expression had a significant interaction with the benefits of PD-1 inhibitor-based therapy in terms of OS (TPS 1%, P = 0.001; TPS 5%, P = 0.037; TPS 10%, P =0.027; CPS 10, P = 0.001) (**Figures 4A, C**). High PD-L1 expression is usually defined as TPS ≥ 1% or CPS ≥ 10, and patients with high PD-L1 expression experienced a better response to PD-1 inhibitor-based therapy than those with low PD-L1 expression.

Subgroup analyses of PD-L1 expression were also performed among different treatment lines and regimens. In general, PD-1 inhibitor-based therapy led to a reduced risk of death compared with chemotherapy regardless of the PD-L1 expression level in those subgroups (**Figures 4B, D**). Patients with high PD-L1 expression experienced a better survival benefit than those with low PD-L1 expression in first-line treatments (CPS 10, P = 0.008; TPS 1%, P = 0.004), second-line treatments (TPS 1%, P = 0.032), immunotherapy (CPS 10, P = 0.007; TPS 1%, P = 0.007) and immunochemotherapy (CPS 10, P = 0.046; TPS 1%, P = 0.041) (**Figures S2–5** and **Figures 4B, D**).

### 3.5 Other clinical features

**Figure 5:**
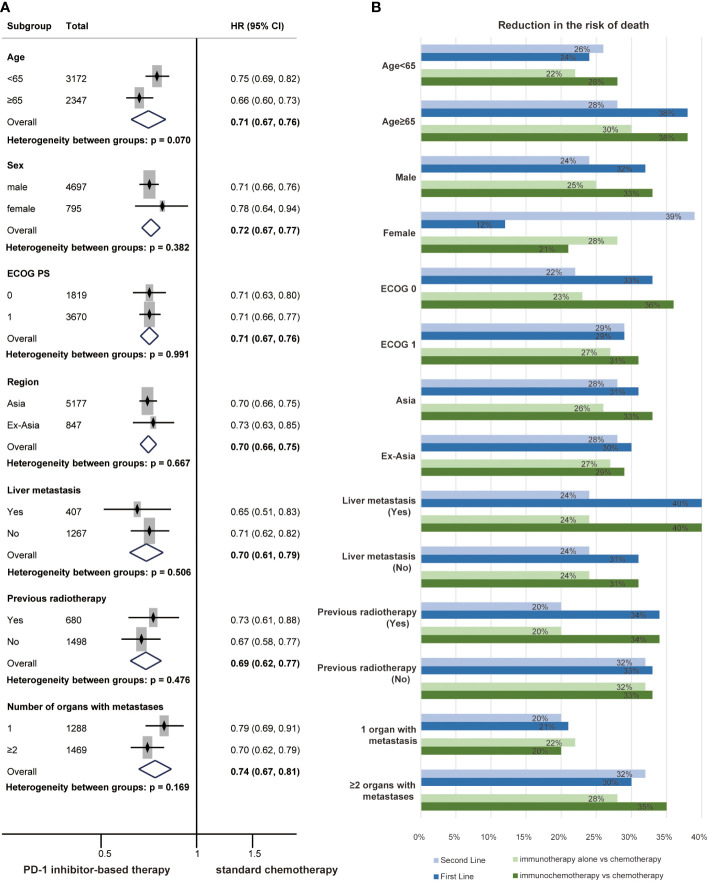
Forest plots of overall survival by subgroup analyses of clinical characteristics **(A)**. The reduced risk of death from PD-1 inhibitor-based therapy by subgroup analyses of clinical characteristics **(B)** in first-line treatments, second-line treatments, immunotherapy and immunochemotherapy.

Subgroup analyses for the risk of death across baseline demographic and disease characteristics were also performed. The HR for OS favoured PD-1 inhibitor-based therapy compared with chemotherapy for all prespecified clinical subgroups, and no significant differences were identified (**Figure 5A**). All subgroups of the patients had a reduced risk of death from PD-1 inhibitor-based therapy in first-line treatments, second-line treatments, immunotherapy and immunochemotherapy (**Figures S2–5** and **Figure 5B**). The results indicated that patients in the subgroup of age ≥ 65 (P = 0.029) or male (P = 0.022) might receive more OS benefit from PD-1 inhibitor-based first-line therapy (**Figure S3**).

### 3.6 Sensitivity analysis and risk of bias

**Figure 6:**
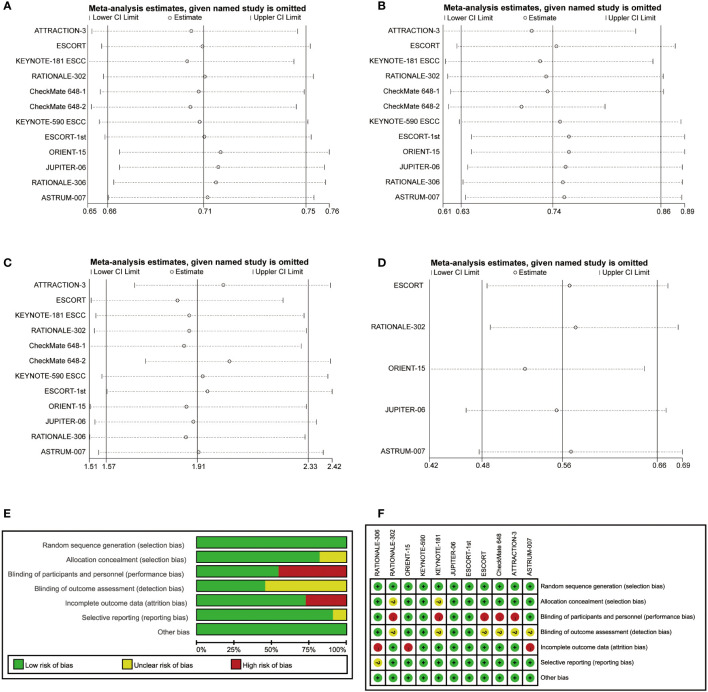
Sensitivity analyses of hazard ratios of overall survival **(A)**, progression-free survival **(B)** and duration of response **(D)** and the odds ratio for objective response rate **(C)**. The risk of bias was evaluated by using Review Manager 5.3 **(E, F)**.

In addition, sensitivity analyses were performed to evaluate the robustness of our findings. The pooled HR for OS, PFS and DOR and the pooled OR for ORR were stable, indicating that the results were not significantly different (**Figures 6A–D**). The bias risk of the included studies was also assessed. All studies were phase 3 RCTs, and the quality assessment rating was relatively high (**Figures 6E, F**).

## 4 Discussion

The process of treating ESCC with PD-1 inhibitors appears to be transitioning from application with later treatment lines to application with earlier treatment lines. ATTRACTION-3 is the first phase 3 study to show that a PD-1 inhibitor (nivolumab) provides a significant OS improvement versus chemotherapy in a global population of patients with metastatic ESCC after one prior therapy (21). KEYNOTE-590 is the first global phase 3 study to confirm that the combination of a PD-1 inhibitor (pembrolizumab) with chemotherapy shows a significant survival benefit in patients with previously untreated, advanced EC (24). Based on KEYNOTE-181 and KEYNOTE-590, the FDA approved pembrolizumab as a second-line treatment for ESCC patients with PD-L1 CPS ≥10 and pembrolizumab plus chemotherapy as a first-line treatment for patients with ESCC. Similarly, based on ATTRACTION-3 and CheckMate 648, the FDA approved nivolumab for patients with pretreated ESCC and nivolumab combination therapy for previously untreated advanced ESCC. In China, combination chemotherapy with a PD-1 inhibitor (pembrolizumab, nivolumab, camrelizumab, sintilimab or toripalimab) has been approved by the NMPA for first-line treatments of advanced ESCC, and PD-1 inhibitor monotherapy (pembrolizumab for CPS ≥10, camrelizumab, nivolumab or tislelizumab) has also been approved for second-line treatments of advanced ESCC. We previously conducted a series of meta-analyses and found that PD-1 inhibitors provided a clinically meaningful survival benefit compared with standard chemotherapy in first-line and second-line treatments of EC (43, 44). However, whether the antitumour activity of PD-1 inhibitors differs between first-line and second-line treatments is unknown. In the present study, we found that PD-1 inhibitor-based therapy improved OS in both first-line and second-line treatments, but there was limited PFS benefit in second-line treatments. Some studies have also shown that the antitumour activity of PD-1 inhibitors is better in earlier lines of therapy than in later lines of therapy (31, 32, 45). This phenomenon may be related to the dynamic changes in the tumour immune microenvironment at different stages of tumour treatment (46).

In recent years, PD-1 inhibitor-based therapeutic regimens have been explored widely in the treatment of ESCC, including PD-1 inhibitor monotherapy, immunochemotherapy or dual immunotherapy (17, 47). We found that the combination of immunotherapy with chemotherapy significantly prolonged OS and PFS. Although immunotherapy alone did not improve PFS, the superior OS suggested that immunotherapy was also a good option for patients with ESCC who were refractory or intolerant to chemotherapy. The TP regimen is recommended as the chemotherapy regimen for patients with ESCC in China, whereas the CF regimen is recommended by the NCCN guidelines in the Ex-Asian region. Different chemotherapy regimens may lead to differences in the immune microenvironment (48). We also evaluated the antitumour activity of PD-1 inhibitors in combination with different chemotherapy regimens for ESCC. Although the benefit of OS appeared to be higher in patients who received PD-1 inhibitors plus TP compared with PD-1 inhibitors plus CF, the difference was not statistically significant. This also indicated that the combination of PD-1 inhibitors and chemotherapy in the treatment of patients with ESCC was generally applicable in different regions.

PD-L1 expression as a biomarker has potentially important predictive value regarding the efficacy of PD-1 inhibitors in a variety of cancers (49, 50). Recent clinical trials of PD-1 inhibitors have generally used immunohistochemistry to evaluate the expression level of PD-L1. The PD-L1 immunohistochemistry kits and antibodies include SP263, 22C3, and 28-8. A study showed that 28-8, 22C3 and SP263 had similar tumour cell positive staining percentages and high consistency (51). There was a trend toward a lower risk of death with PD-1 inhibitors regardless of the PD-L1 expression level. However, the HR for OS in patients with high PD-L1 expression was less than that in patients with low PD-L1 expression, and the difference was statistically significant, which suggested that the PD-L1 expression level can be used as a predictor of survival benefit from PD-1 inhibitor therapy in advanced ESCC.

Previous studies have shown that the efficacy of PD-1 inhibitors may be related to clinical characteristics such as region and ECOG PS (33, 52). Patients with advanced ESCC were grouped according to clinical and pathological characteristics, and subgroup analyses were performed. The results showed that PD-1 inhibitor-based therapy significantly prolonged OS across all subgroups. Although we did not distinguish a dominant population that would benefit from PD-1 inhibitors in subgroups of clinical characteristics, we showed that PD-1 inhibitors have general applicability in patients with ESCC.

Moreover, subgroup analyses of PD-L1 expression and clinical characteristics were also performed in the first-line, second-line, immunotherapy and immunochemotherapy populations. Similarly, subgroup analyses revealed a lower risk of death in patients with PD-1 inhibitor-based therapy compared to patients with chemotherapy. Patients in all subgroups experienced OS benefits from PD-1 inhibitor-based therapy versus chemotherapy, indicating that PD-1 inhibitors have common applicability in the treatment of patients with advanced ESCC. High PD-L1 expression populations also dominated among the patients who would benefit from PD-1 inhibitors. Furthermore, the risk of death was significantly lower in patients receiving first-line treatments who were older than 65 years or male; this finding was consistent with some studies (53, 54). The ageing process can influence the proportion of immune cells, resulting in an increase in T-cell-mediated immune responses. Sex-specific differences in the immune microenvironment might explain the differences in survival benefits (55). Regardless, this difference was not found for second-line treatments, and the roles of age and sex in the immunotherapy of ESCC need to be explored further.

Our meta-analysis has the following advantages. All included studies were large phase 3 clinical trials, and all data were the latest, indicating that the results of this meta-analysis are reliable. We carried out detailed subgroup analyses according to therapeutic, pathological and clinical characteristics in patients with ESCC, which was novel and meaningful. There are some limitations in this meta-analysis. The PD-L1 antibodies of immunohistochemical staining are different, and subgroup analyses according to PD-L1 testing methods are not available because of the limitation in the number of clinical trials. We can continue to pay attention to such progress in advanced ESCC. Our finding that patients with high PD-L1 expression may derive a greater benefit from PD-1 inhibitor treatment than those with low PD-L1 expression will require validation in further prospective clinical trials.

## 5 Conclusions

Compared with standard chemotherapy, PD-1 inhibitor-based therapy led to a significantly better survival benefit in patients with ESCC. Immunochemotherapy provided a higher PFS benefit than immunotherapy alone. PD-1 inhibitor-based therapy exhibited OS benefits according to subgroup analyses of treatment lines, treatment regimens and clinical characteristics, suggesting that PD-1 inhibitors have common applicability in the treatment of patients with advanced ESCC. Survival benefits were better in patients with high PD-L1 expression than in those with low PD-L1 expression, suggesting that the PD-L1 expression level can be used as a predictor of survival benefit from PD-1 inhibitor therapy.

## Data availability statement

The raw data supporting the conclusions of this article will be made available by the authors, without undue reservation.

## Author contributions

YL: Literature searching, Data extraction, Literature screening, Statistical analysis, Assessment of study quality, Writing and Original Draft. WW: Literature searching, Data extraction, Literature screening, Assessment of study quality. FW: Statistical analysis, Writing-Review and Editing, Funding acquisition. All authors contributed to the article and approved the submitted version.
