# Supplementary material for: Clinical benefits of PD-1 inhibitors in specific subgroups of patients with advanced esophageal squamous cell carcinoma: a systematic review and meta-analysis of phase 3 randomized clinical trials
Source: Front Immunol. 2023 May 2;14:1171671. doi: 10.3389/fimmu.2023.1171671 (PMC10185849; doi:10.3389/fimmu.2023.1171671)
Supplement: Supplementary file 1 [file DataSheet_1.pdf]

frontiers

Supplementary Material

Clinical benefits of PD-1 inhibitors in specific subgroups of patients with advanced esophageal squamous cell carcinoma: A systematic review and meta-analysis of phase 3 randomized clinical trials

Yao Lu[1], Wenkang Wang[2], Feng Wang[1*]

*** Correspondence:** Feng Wang, Email: zzuwangfeng@zzu.edu.cn；

1 Supplementary Figures and Tables

1.1 Supplementary Figures

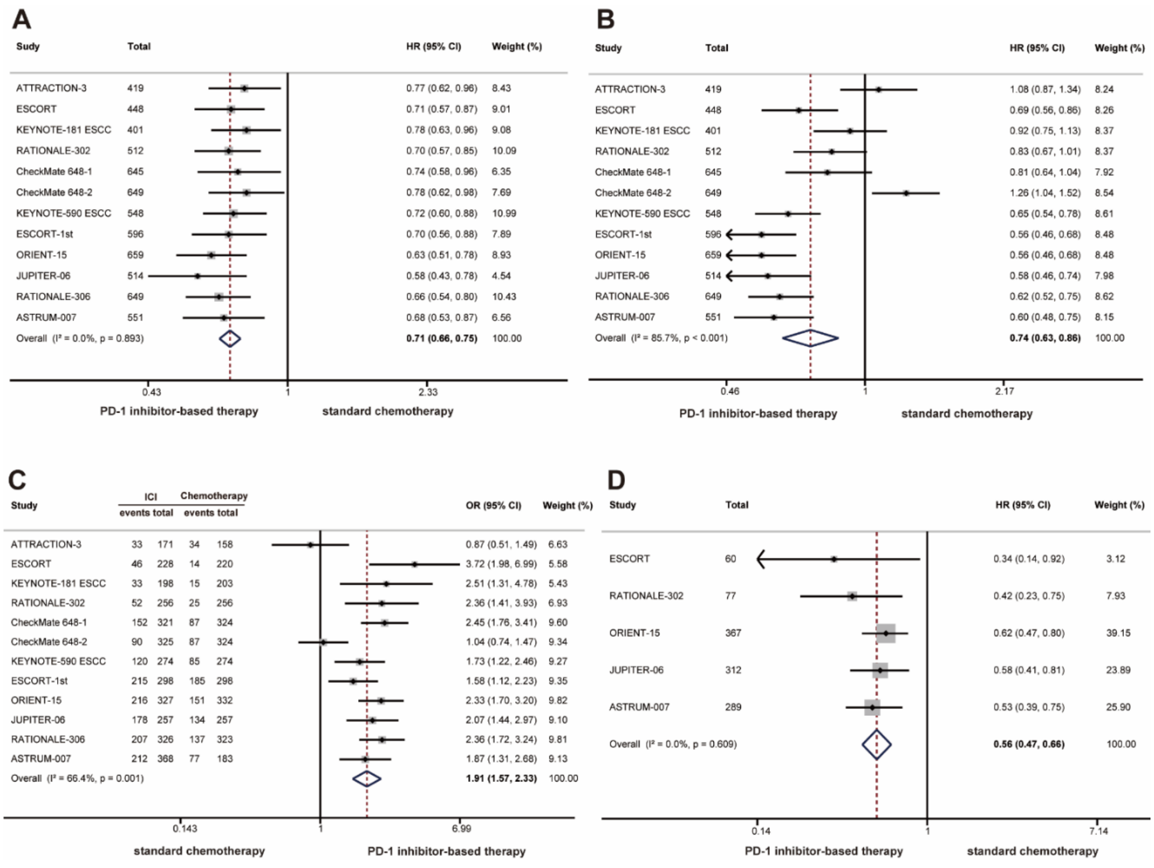

Supplementary Figure 1. Pooled hazard ratios for overall survival (A), progression-free survival (B), duration of response (D), and pooled odds ratio for objective response rate (C) in advanced esophageal squamous cell carcinoma treated with PD-1 inhibitor-based therapy versus chemotherapy. (HR: hazard ratio; OR: odds ratio; CI: confidence interval; PD-1: programmed cell death 1).

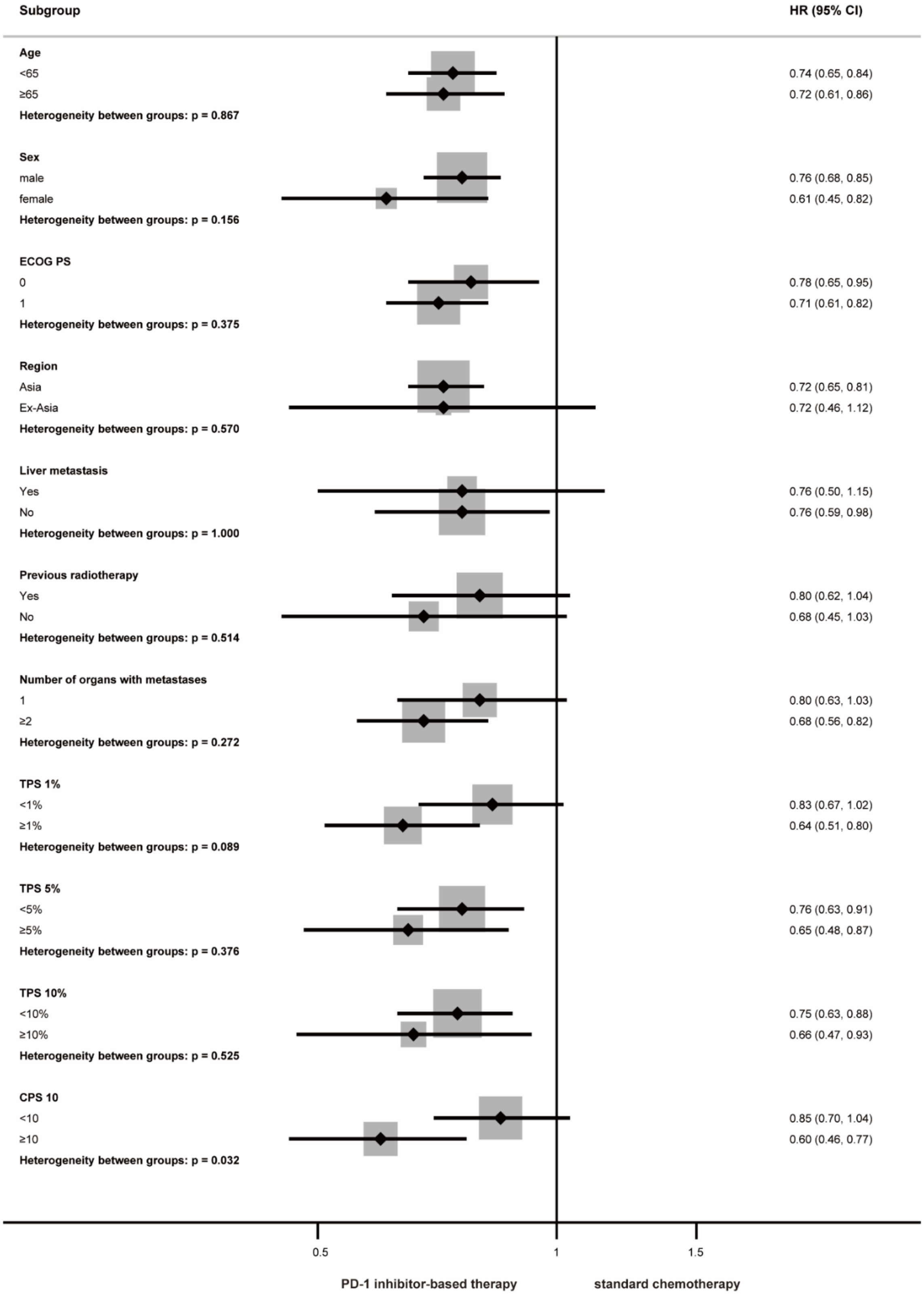

Supplementary Figure 2. Forest plots of overall survival by subgroup analyses of PD-L1 expression and clinical characteristics in second-line treatments. (HR: hazard ratio; CI: confidence interval; TPS: tumour proportion score; CPS: combined positive score).

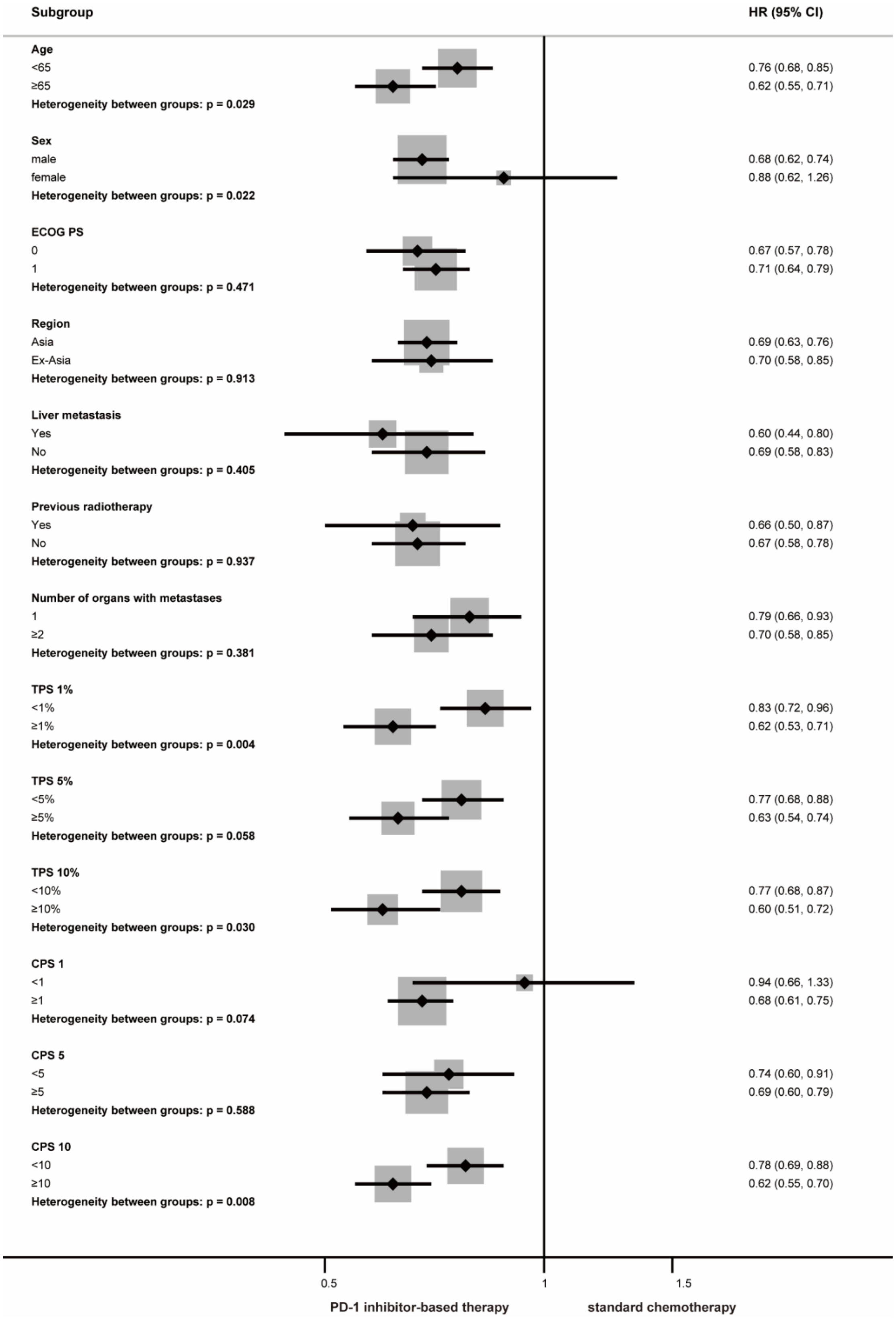

Supplementary Figure 3. Forest plots of overall survival by subgroup analyses of PD-L1 expression and clinical characteristics in first-line treatments. (HR: hazard ratio; CI: confidence interval; TPS: tumour proportion score; CPS: combined positive score).

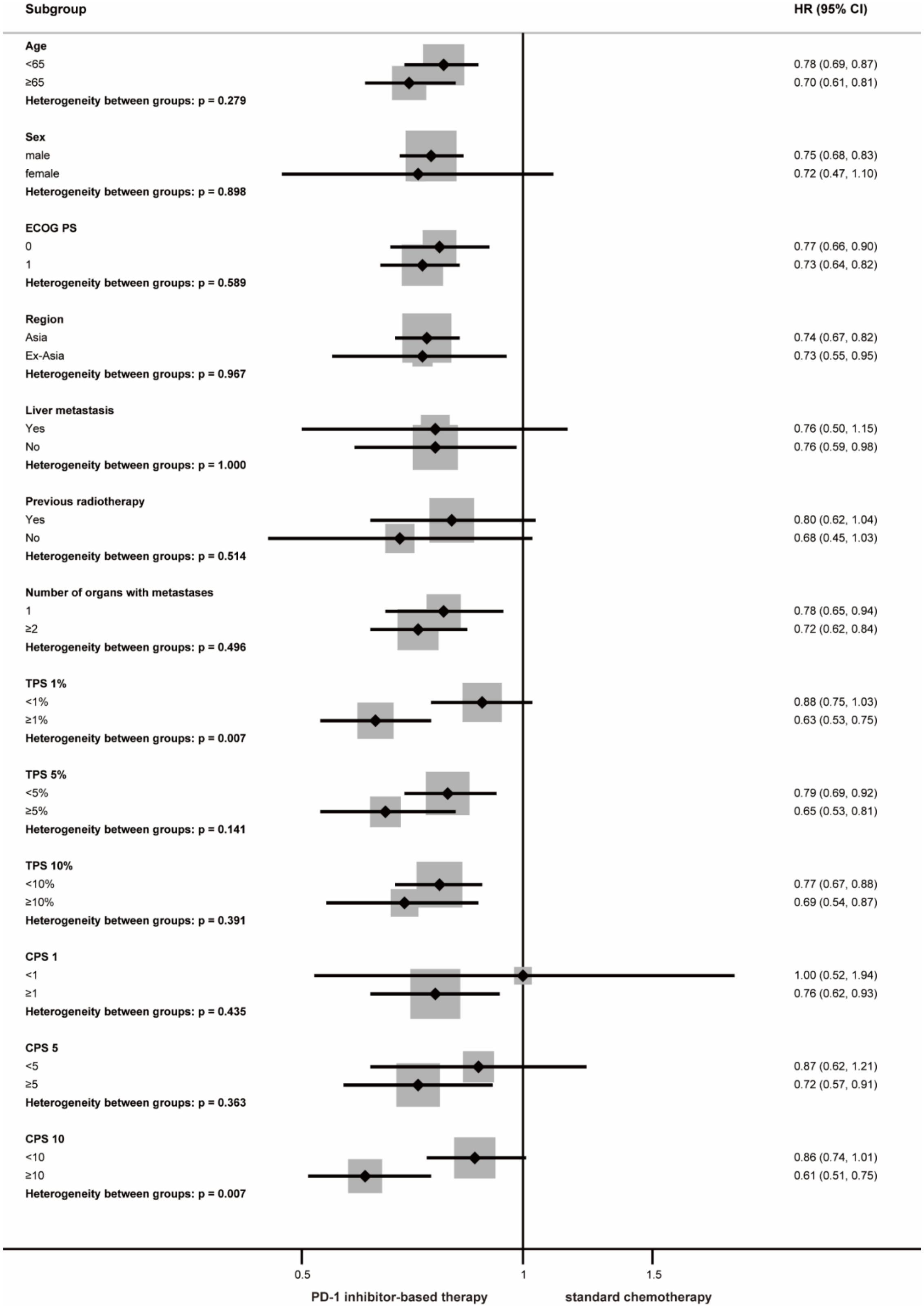

Supplementary Figure 4. Forest plots of overall survival by subgroup analyses of PD-L1 expression and clinical characteristics in immunotherapy. (HR: hazard ratio; CI: confidence interval; TPS: tumour proportion score; CPS: combined positive score).

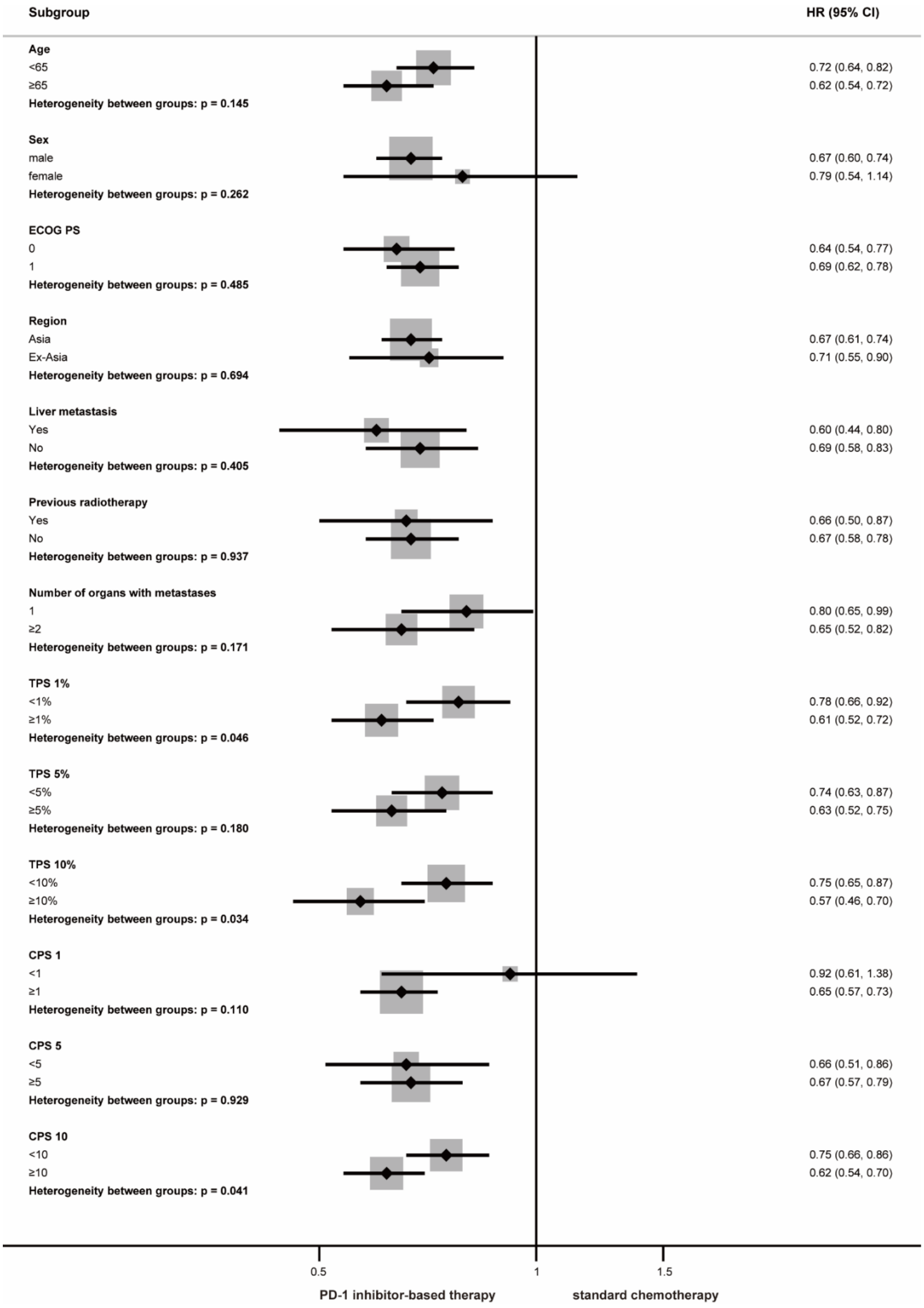

Supplementary Figure 5. Forest plots of overall survival by subgroup analyses of PD-L1 expression and clinical characteristics in immunochemotherapy. (HR: hazard ratio; CI: confidence interval; TPS: tumour proportion score; CPS: combined positive score).

1.2 Supplementary Table 1

Supplementary Table 1. Analyses were performed for PD-L1 immunohistochemical antibodies and the proportion of PD-L1 positive patients.

Study	PD-1 inhibitor	PD-L1 CPS>10		PD-L1 TPS>1		IHC kit/ab	Line
		N	%	N	%		
ATTRACTION-3	nivolumab	-	-	203	48.45%	28-8	2
CheckMate 648-1	nivolumab	280	45.98%	314	48.83%	28-8	1
CheckMate 648-2	nivolumab	271	45.09%	314	48.76%	28-8	1
KEYNOTE-181 ESCC	pembrolizumab	167	42.28%	-	-	22C3	2
KEYNOTE-590 ESCC	pembrolizumab	143	53.16%	-	-	22C3	1
ORIENT-15	sintilimab	381	57.81%	362	54.93%	22C3	1
ESCORT	camrelizumab	-	-	191	43.61%	6E8	2
ESCORT-1st	camrelizumab	-	-	329	56.24%	6E8	1
JUPITER-06	toripalimab	212	43.44%	-	-	JS311	1
RATIONALE-302	tislelizumab	157	38.01%	-	-	SP263	2

Abbreviations: IHC: immunohistochemistry; ab: antibody; N: number; %: percentage.
